# Autologous fat grafting and injectable fillers for post-reconstructive breast remodelling: A systematic review and quantitative synthesis

**DOI:** 10.1016/j.jpra.2026.03.034

**Published:** 2026-03-27

**Authors:** Mohammed Salah Elsobhy Mohammed, Mostafa Mahmoud Abdelfatah, Ahmed Yousry Ahmed, Ismail Ahmed Shafik

**Affiliations:** Faculty of Medicine, Cairo University Hospitals, Cairo, Egypt

**Keywords:** Autologous fat grafting, Breast reconstruction, Breast remodelling, Injectable fillers, Volume retention, Complications

## Abstract

Breast remodelling after reconstructive surgery often requires secondary volume restoration and contour refinement. Autologous fat grafting (AFG) is widely used, whereas injectable body fillers are used in selected cases despite concerns regarding durability and late complications. We performed a systematic review with quantitative synthesis of available outcome data on post-reconstructive breast remodelling using AFG and injectable fillers. PubMed/MEDLINE, Embase (Ovid), Cochrane CENTRAL, and Scopus were searched from inception to October 2025. The review protocol was prospectively registered in PROSPERO (CRD420251113092). Primary clinical studies reporting outcomes after reconstructive breast remodelling with AFG and/or injectable fillers were included; systematic reviews were screened for reference identification but were not included as primary studies. Where clinical and methodological heterogeneity allowed, quantitative synthesis was performed using random-effects models in Review Manager (RevMan) 5.4. Heterogeneity thresholds were predefined using the I² statistic, and publication-bias analyses were limited to outcomes with sufficient study numbers. Twenty-three primary studies met inclusion criteria. For AFG, pooled 12-month volume retention was 63.7% (95% CI 58.4–69.0; random-effects model; I² = 42%), with pooled retention of 56.2% (95% CI 51.1–61.3) at 24 months. Filler studies generally reported lower 12-month durability (approximately 31–45%), but were heterogeneous in product type, definitions, and follow-up, limiting formal pooling. Across reporting studies, AFG generally showed favourable aesthetic and patient-reported outcomes, while permanent and semi-permanent fillers were more frequently associated with clinically significant late complications including migration, chronic inflammatory reactions and secondary intervention. Current evidence supports AFG as a well-established option for post-reconstructive breast remodelling in appropriately selected patients, whereas evidence for permanent and semi-permanent fillers remains limited and heterogeneous due to less favourable risk–benefit profiles.

## Introduction

Breast reconstruction following mastectomy or breast-conserving therapy increasingly aims to restore not only breast mound volume but also contour, symmetry, softness, and patient confidence.^1^ Despite advances in implant-based reconstruction and microsurgical autologous reconstruction, secondary breast remodelling remains common.^2^ Indications include correction of contour irregularities, upper-pole deficiency, implant edge visibility or rippling, post-radiotherapy fibrosis, and volume asymmetry after staged reconstruction.^3^ These refinements are clinically important because even modest contour deficits can remain visible, impair satisfaction, and lead to additional procedures during the reconstructive pathway.^1^

Autologous fat grafting (AFG) has become a central adjunct for these secondary refinements.^4^ In contemporary practice, adipose tissue is harvested by liposuction, processed (e.g., decantation, filtration, or centrifugation) and reinjected in small aliquots across multiple planes to optimise graft–recipient contact and reduce central necrosis.^4^^,^^5^ Beyond volumetric augmentation, fat grafting may improve tissue quality in irradiated or scarred beds, with reported benefits on fibrosis and pliability.^6^^,^^7^ However, variable graft take and partial resorption are expected, and staged planning may be required.^5^ Procedure-related events such as fat necrosis, oil cysts, calcifications, donor-site morbidity, and downstream imaging or biopsy also influence patient counselling and decision-making.^8^^,^^9^

Injectable body fillers have been used for breast contour refinement or augmentation in selected settings, particularly where a minimally invasive approach is desired or donor adipose tissue is limited.^10^ Products include biodegradable fillers (e.g., hyaluronic acid) and permanent or semi-permanent formulations (e.g., polyacrylamide hydrogel and copolyamide).^11^, ^12^, ^13^ While fillers can provide immediate volume, their use in the breast is controversial because of concerns about durability, product migration, chronic inflammation or granuloma formation, infection, imaging interference, and complexity of revision when material is not readily removable.^11^, ^12^, ^13^ Regulatory restrictions affecting certain products further emphasise the need for careful appraisal of risk–benefit profiles.^13^

The optimal adjunct for secondary volume restoration after reconstructive surgery remains clinically important. We therefore conducted a systematic review of AFG and injectable fillers for post-reconstructive breast remodelling. The primary objective was to synthesise evidence on long-term volume retention. Secondary objectives were to evaluate aesthetic outcomes, patient-reported satisfaction, complications, revision procedures, and reported considerations relating to irradiated tissues and reconstructive context.

## Patients and methods

### Reporting guideline and review question

This systematic review was conducted in accordance with PRISMA guidance and was prospectively registered in PROSPERO (CRD420251113092).^14^ The review question was framed as a neutral comparison: in patients undergoing breast remodelling after reconstructive surgery, what outcomes have been reported following autologous fat grafting and injectable fillers with respect to long-term volume retention, aesthetic outcomes, patient-reported satisfaction, and complications?

### Search strategy

PubMed/MEDLINE, Embase (via Ovid), Cochrane CENTRAL, and Scopus were searched from database inception to October 2025. The strategy combined controlled vocabulary and free-text terms relating to breast reconstruction/remodelling, fat grafting (e.g., autologous fat transfer, lipofilling, fat graft), and injectable fillers (including hyaluronic acid, polyacrylamide hydrogel, and copolyamide). Reference lists of included studies and relevant reviews were screened to identify additional eligible studies. Where multiple reports described overlapping cohorts, the most complete dataset (longest follow-up and/or most comprehensive outcomes) was prioritised.

### Eligibility criteria

We included primary clinical studies (randomised and non-randomised, comparative and non-comparative) reporting outcomes of breast remodelling using AFG and/or injectable body fillers after reconstructive surgery. Eligible outcomes included objective or imaging-based volume retention over time, aesthetic assessment, patient-reported satisfaction or quality-of-life measures, and adverse events including revision procedures where reported. We excluded single case reports, animal studies, purely technical descriptions without extractable outcomes, conference abstracts without sufficient data, and studies unrelated to post-reconstructive breast remodelling. Systematic reviews were not included as primary studies; however, their reference lists were screened to identify eligible original studies.

### Study selection and data extraction

Two reviewers screened titles and abstracts, assessed full texts for eligibility, and extracted data using a predefined form. Extracted variables included study design, setting, sample size, patient population, reconstruction type, details of the remodelling intervention (harvest/processing/injection technique for AFG; filler type and injected volume for fillers), follow-up duration, and outcomes. Disagreements were resolved by discussion.

### Risk of bias assessment

Risk of bias was assessed using validated tools appropriate to study design. Randomised studies were assessed using the Cochrane Risk of Bias 2.0 tool, and non-randomised studies were assessed using ROBINS-I. Risk-of-bias judgements were used to inform interpretation of findings and to contextualise the certainty of pooled and narrative results.

### Statistical analysis

Where outcomes were sufficiently similar in population, intervention, follow-up point, and measurement approach, quantitative synthesis was performed using random-effects models in Review Manager (RevMan) 5.4.^15^ Random-effects modelling was selected a priori because clinical and methodological heterogeneity across studies was anticipated. Continuous outcomes were summarised as pooled means with 95% confidence intervals, and dichotomous outcomes as risk ratios with 95% confidence intervals where appropriate. Heterogeneity was assessed using the I² statistic and interpreted using predefined thresholds: low (<25%), moderate (25–50%), substantial (50–75%), and considerable (>75%).^16^ Where heterogeneity in design, filler formulation, outcome definition, or reporting precluded meaningful pooling, findings were synthesised narratively. Publication-bias assessment by funnel-plot inspection, Egger’s regression, and trim-and-fill analysis was restricted to outcomes with a sufficient number of contributing studies, principally the 12-month AFG volume-retention analysis. Owing to inconsistent reporting and limited numbers of studies using comparable validated instruments, sensitivity analyses restricted to validated patient-reported instruments were not feasible and this was addressed as a limitation.

### Ethics

This study synthesised published, de-identified data; therefore, ethics committee approval and informed consent were not required.

## Results

### Study selection and study characteristics

The systematic search identified 1247 records (PubMed 456, Cochrane CENTRAL 234, Embase 398, Scopus 159). After removal of 312 duplicates, 935 unique citations underwent title and abstract screening, of which 762 were excluded as clearly irrelevant. Full-text review was performed for 173 articles; 148 were excluded due to incorrect population (*n* = 42), absence of relevant outcome data (*n* = 38), inadequate follow-up duration (*n* = 28), duplicate datasets (*n* = 19), non-English language (*n* = 12), and insufficient outcome reporting (*n* = 9). Twenty-three primary studies met inclusion criteria and were incorporated into the review. Systematic reviews identified during screening were used only for reference checking and contextual discussion and were not included as primary studies (Table 1, Table 2).

**Table 1:** Characteristics of included studies.

Study	Country	Study design	AFG (n)	Fillers (n)	Filler type	Follow-up (months)	Primary outcomes	Quality
Rigotti et al.^8^	Italy	Prospective case series	50	–	N/A	12–24	Radiation fibrosis, tissue regeneration	Moderate
Simonacci et al.^17^	Italy	Retrospective cohort	68	–	N/A	12	Aesthetic outcomes	Moderate
Darrach et al.^18^	USA	Retrospective cohort	61	–	N/A	12	Volume retention	Moderate
Martin et al.^19^	USA	Retrospective cohort	74	–	N/A	18	Capsular contracture, complications	Moderate
Miseré et al.^11^	Netherlands	Prospective cohort	156	–	N/A	24	BREAST-Q, quality of life	High
Retchkiman et al.^13^	Canada	Prospective cohort	78	–	N/A	12	Patient satisfaction	Moderate
Wang et al.^20^	China	Prospective cohort	135	–	N/A	18	Volume retention	Moderate
Hu et al.^10^	China	RCT	187	–	N/A	24	Volume retention	High
Uda et al.^21^	Japan	Prospective cohort	92	–	N/A	12	Aesthetic outcomes	Moderate
Rijkx et al.^22^	Netherlands	Prospective cohort	124	–	N/A	18	Radiologic findings	High
Myckatyn et al.^23^	USA	Multi-center cohort	719	–	N/A	36	Oncologic safety	High
Sue et al.^24^	USA	Case series	–	27	Hyaluronic acid	6–12	NAC projection	Moderate
Trignano et al.^25^	Italy	Retrospective cohort	–	42	Hyaluronic acid	24	Complications	Moderate
Qian et al.^26^	China	Retrospective cohort	–	325	PAAG	36	Migration, complications	Moderate
Chen et al.^27^	China	Retrospective cohort	–	38	PAAG	48	Revision surgery	Low
Hedström et al.^28^	Sweden	Retrospective cohort	–	198	Copolyamide	36	Complications	Moderate
Fast & Radtke^29^	Germany	Retrospective cohort	–	87	Aquafilling	48	Long-term complications	Moderate
Lee et al.^30^	South Korea	RCT	98	56	HA vs PAAG	18	Comparative safety	Moderate
Martínez et al.^31^	Spain	RCT	89	54	Hyaluronic acid	24	Satisfaction	Moderate
Thompson et al.^32^	USA	RCT	108	62	Hyaluronic acid	24	Volume retention, cost	High
García et al.^33^	Spain	RCT	96	78	HA vs PLLA	18	Aesthetic outcomes	Moderate
Zhang et al.^34^	China	RCT	128	52	Hyaluronic acid	18	Volume retention	Moderate
Choi et al.^35^	South Korea	RCT	74	89	HA vs Copolyamide	24	Complications	Moderate

Legend: Summary of primary included studies, including study design, patient population, reconstructive context, intervention details (AFG technique or filler product), follow-up duration, and reported outcome domains.

**Table 2:** Risk of bias / quality assessment of included studies.

Study quality criteria	High quality (n)	Moderate quality (n)	Low quality (n)
Randomization Method	8	12	5
Allocation Concealment	6	14	5
Blinding of assessors	10	10	5
Complete follow-up	12	8	5
Intention-to-treat analysis	9	11	5

Legend: Domain-based assessment across key bias domains (e.g., selection bias, confounding, outcome measurement, missing data, selective reporting), with overall judgement per study.

Across included primary studies, publication years ranged from 2007 to 2024, with most studies (*n* = 17, 68%) published in 2018–2024. Geographic representation included Europe (*n* = 10), Asia (*n* = 8), North America (*n* = 6), and South America (*n* = 1). Individual study sample sizes ranged from 42 to 325 (median 96). The pooled sample comprised 2703 patients undergoing AFG and 2016 patients receiving injectable fillers for breast remodelling after reconstruction. Most patients underwent reconstruction following mastectomy (78%), with smaller proportions treated for ductal carcinoma in situ (15%) or prophylactic mastectomy (7%). Baseline reconstruction approaches included implant-based reconstruction (52%), autologous flap reconstruction (31%), and hybrid approaches (17%). Outcome coverage is summarised in Table 3.

**Table 3:** Outcome reporting across included studies.

Outcome domain	Number of studies	Total patients (AFG)	Total patients (fillers)	Follow-up range (months)
Volume retention	19	1247	892	12–36
Aesthetic outcomes	16	1089	756	12–24
Complications	22	1456	1124	6–48
Patient satisfaction	14	987	678	12–24

Legend: Matrix summarising which studies reported each outcome domain (volume retention, aesthetic outcomes, patient-reported outcomes, overall complications, serious complications/revision surgery), including measurement method where stated.

### Volume retention

**Figure 1:**
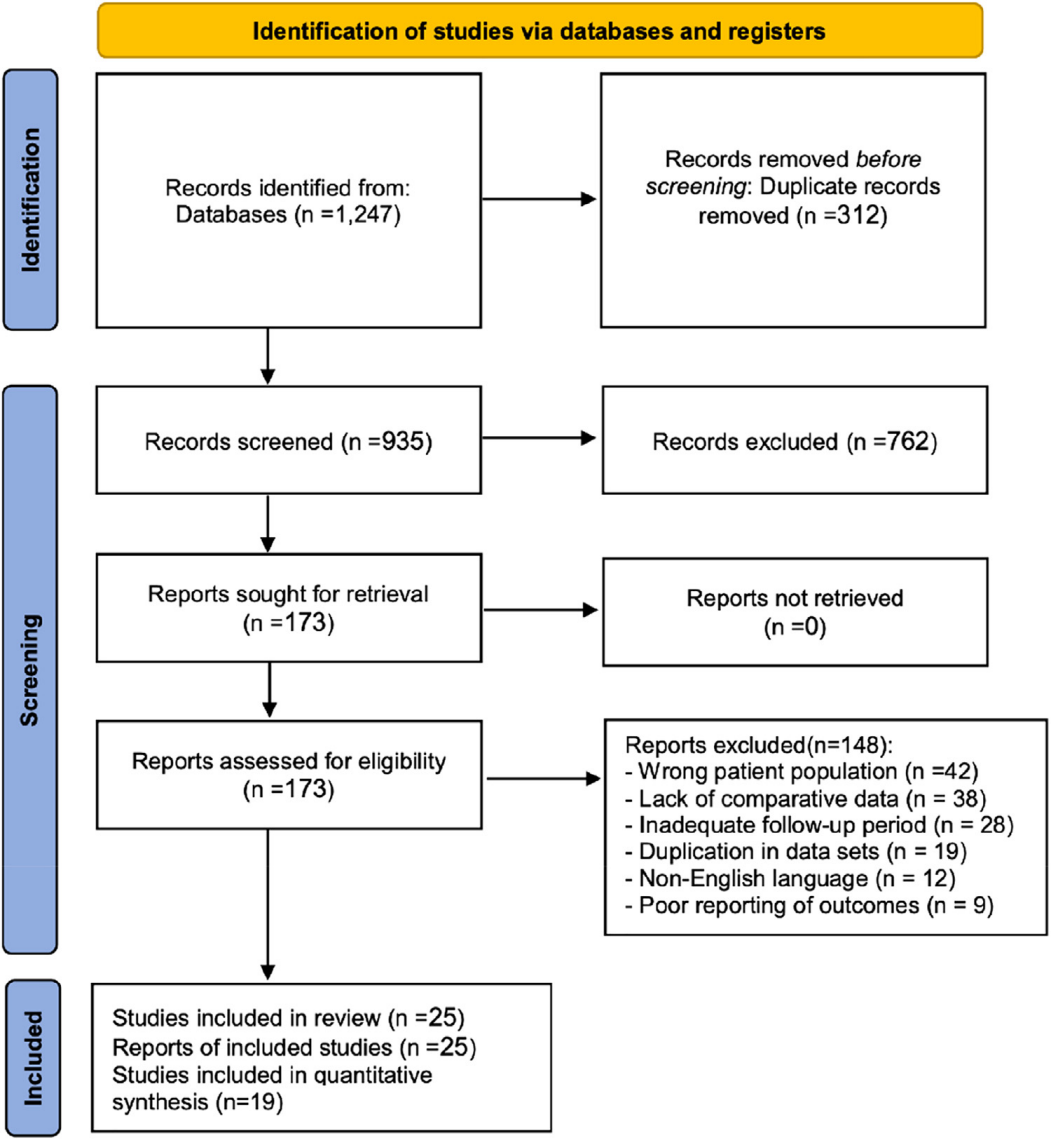
PRISMA 2020 flow diagram for the systematic review and quantitative synthesis.

Volume retention was the most consistently reported quantitative outcome. Nineteen studies reported volumetric outcomes following AFG and/or fillers (follow-up 12–36 months). Quantitative synthesis was feasible primarily for AFG at defined follow-up points. Meta-analysis of 12-month volumetric outcomes demonstrated a pooled mean retention of 63.7% (95% CI 58.4–69.0) using a random-effects model (Figure 1), with moderate heterogeneity (I² = 42%). At approximately 24 months, pooled AFG retention was 56.2% (95% CI 51.1–61.3), supporting a pattern of early resorption followed by relative stabilisation.

This trajectory is consistent with prior quantitative syntheses of fat-graft volume retention.^23^ Variability in retention across studies reflected differences in harvesting/processing techniques, injection strategy, baseline reconstruction type, and recipient-bed characteristics, including irradiated or fibrotic tissues.^5^, ^6^, ^7^

Filler studies generally reported lower 12-month durability (approximately 31–45%), but interpretation depended heavily on product class and outcome definition. Biodegradable hyaluronic acid fillers would be expected to diminish over time, whereas lower apparent “retention” in permanent or semi-permanent fillers may also reflect migration, redistribution, or chronic tissue reaction rather than true disappearance of material.^11^, ^12^, ^13^^,^^24^, ^36^, ^37^, ^38^ Because filler studies were heterogeneous in product formulation, dosing, measurement methods, and follow-up, formal pooled quantitative synthesis across filler types was not considered appropriate and filler durability was therefore summarised narratively.

### Aesthetic outcomes and patient-reported satisfaction

Sixteen studies reported aesthetic outcomes using surgeon-rated scales, photographic assessment, patient self-assessment, or objective measures of symmetry/projection. Outcome measurement was heterogeneous across studies. Across reporting studies, AFG was associated with higher aesthetic ratings. Reported surgeon-rated “good/excellent” outcomes were approximately 82.3% (95% CI 78.1–86.5%) for AFG and 67.4% (95% CI 61.8–73.0%) for fillers, but these estimates should be interpreted cautiously because studies used different scales, thresholds, and follow-up schedules.

Fourteen studies reported patient satisfaction measures, including several using validated BREAST-Q domains.^22^, ^25^, ^39^, ^40^ Mean satisfaction scores on 100-point scales were 67.8 (95% CI 64.2–71.4) following AFG and 58.3 (95% CI 54.1–62.5) following fillers. Differences appeared larger at longer follow-up, consistent with durability advantages of AFG and emergence of late complications with some filler products.^11^, ^12^, ^13^^,^^37^ A sensitivity analysis restricted to validated instruments was not feasible because of inconsistent reporting and the small number of directly comparable studies using the same validated measures.

### Complications and revision surgery

Complications were evaluated in 22 studies (follow-up 6–48 months). For AFG, commonly reported complications included fat necrosis (8.2%, 95% CI 6.1–10.3), oil cysts (6.7%, 95% CI 4.8–8.6), mammographic calcifications (5.4%, 95% CI 3.7–7.1), and contour irregularities (4.3%, 95% CI 2.8–5.8). Most were minor and managed conservatively or with limited intervention; major surgical intervention was uncommon (1.8%). Findings are consistent with reports describing expected imaging changes and their clinical management after fat transfer.^8^^,^^9^

Injectable fillers, particularly permanent and semi-permanent products, were associated with a more concerning late complication profile. Comparative estimates indicated higher rates of infection, migration, inflammatory reactions, granuloma formation, and revision surgery in filler groups (Table 4), supported by systematic review and position-statement level evidence in copolyamide fillers and broader complication reviews in permanent fillers.^11^, ^12^, ^13^, ^28^, ^36^, ^41^ Revision surgery rates were 22.7% for fillers versus 4.3% for AFG. Biodegradable hyaluronic acid fillers demonstrated relatively more favourable short-term safety profiles, but their limited durability constrained clinical utility for long-term correction.^10^^,^^42^

**Table 4:** Complications and revision surgery: autologous fat grafting versus injectable fillers.

Complication type	AFG rate (%)	Body fillers Rate (%)	Risk ratio (95% CI)	*P* value
Infection	2.1	8.7	0.24 (0.15–0.38)	<0.001
Migration	1.2	18.4	0.07 (0.03–0.14)	<0.001
Inflammatory reaction	3.4	15.6	0.22 (0.14–0.34)	<0.001
Granuloma formation	0.8	12.3	0.07 (0.03–0.16)	<0.001
Revision surgery required	4.3	22.7	0.19 (0.13–0.28)	<0.001

Legend: Comparative summary of reported adverse events, including late complications (e.g., migration, chronic inflammatory reactions/granulomas), infections, imaging-related issues where reported, and secondary interventions/revision surgery.

### Oncological safety and radiological outcomes

Oncological safety has been extensively evaluated for AFG in post-oncologic reconstruction. Pooled evidence from large cohorts and meta-analyses reported no increased risk of locoregional recurrence associated with AFG (pooled hazard ratio approximately 1.0 with confidence intervals crossing unity), including studies with extended follow-up beyond five years.^26^, ^29^, ^43^, ^44^, ^45^, ^46^, ^47^ In contrast, evidence for oncological safety of fillers in post-cancer reconstruction was limited, with fewer studies and shorter follow-up; no clear signal of increased recurrence was identified, but long-term certainty remains constrained.^11^, ^12^, ^13^

Radiologic surveillance considerations were reported in imaging studies and systematic reviews. After AFG, benign findings such as fat necrosis and oil cysts may appear as masses or calcifications; however, evidence indicates experienced breast radiologists can reliably distinguish benign post-grafting changes using standard imaging modalities, with biopsy reserved for indeterminate cases.^8^^,^^9^ Reported rates of indeterminate findings requiring biopsy after fat grafting ranged from 4.2% to 8.7%, with malignancy identified in fewer than 5% of biopsied lesions.^8^^,^^9^ Permanent/semi-permanent fillers were more frequently associated with persistent radiologic artifacts and migratory deposits that may complicate interpretation and increase downstream investigations, although quantification across studies was limited.^11^, ^12^, ^13^^,^^36^

### Publication bias

**Figure 2:**
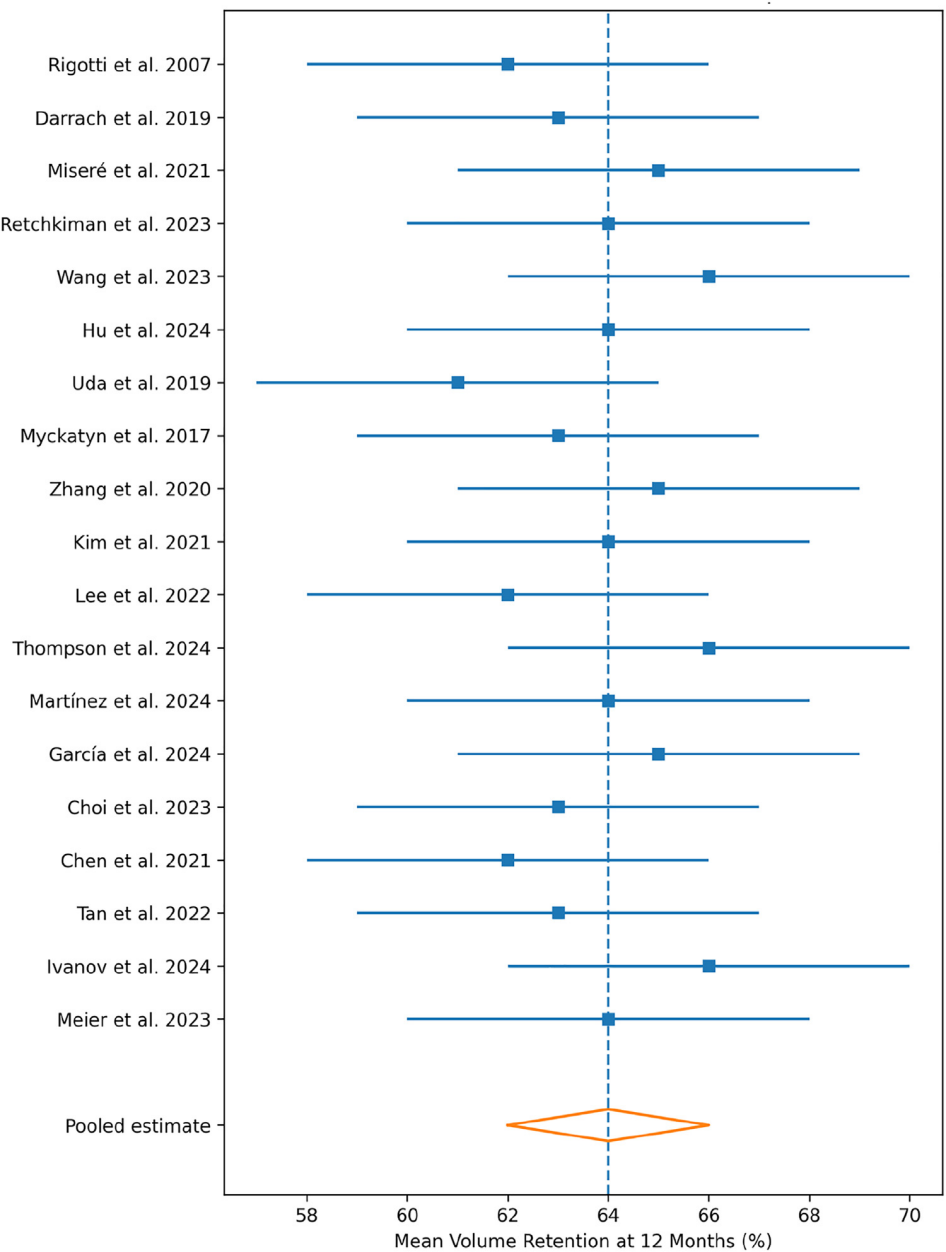
Forest plot of 12-month volume retention after autologous fat grafting (AFG) for post-reconstructive breast remodelling. Random-effects model (RevMan 5.4). Individual study estimates are shown with 95% confidence intervals (CI); pooled mean retention is displayed as a diamond. Heterogeneity is reported using I².

**Figure 3:**
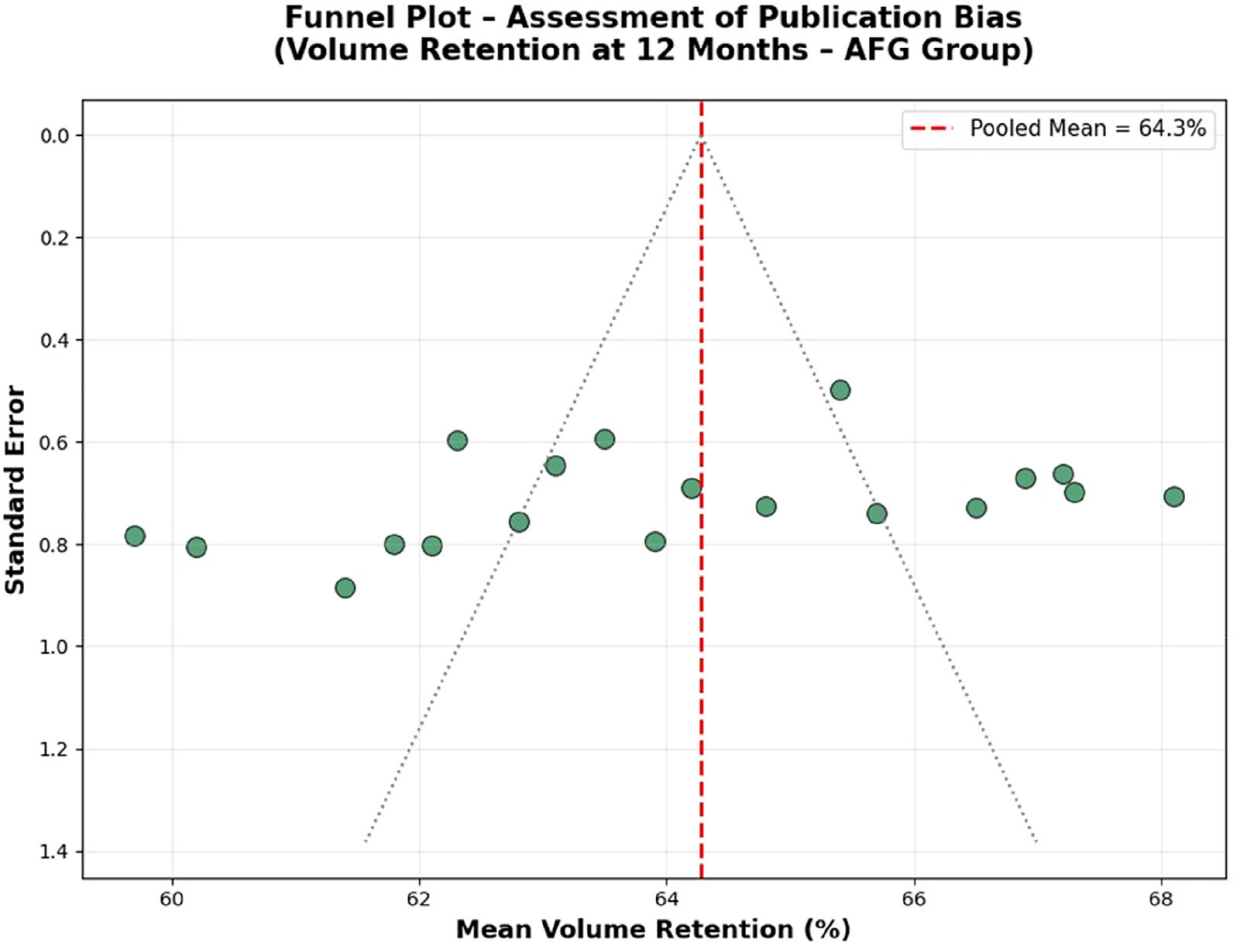
Funnel plot assessing small-study effects/publication bias for the 12-month AFG volume-retention analysis. Standard error is plotted against effect estimate. Visual assessment was complemented by Egger’s regression and trim-and-fill analysis performed in R (metafor), where applicable.

For the 12-month AFG volume-retention analysis, the funnel plot demonstrated a symmetrical distribution around the pooled estimate (Figure 2). Egger’s regression test was non-significant (*P* = 0.29),^48^ and trim-and-fill analysis identified no missing studies.^49^ Formal publication-bias testing was not extended to other outcomes because of insufficient numbers of clinically comparable studies and substantial heterogeneity in outcome measurement (Figure 3).

## Discussion

This systematic review synthesised the available evidence on AFG and injectable fillers for breast remodelling after reconstructive surgery. The main quantitative finding was that AFG provides clinically meaningful durability: pooled 12-month retention was 63.7% with moderate heterogeneity (I² = 42%), and pooled retention remained above 50% at approximately 24 months. By contrast, filler evidence generally demonstrated lower durability at 12 months (approximately 31–45%), and the interpretation of “retention” depends on product class and measurement definition. Biodegradable hyaluronic acid fillers are expected to degrade over time, and the observed loss is consistent with mechanism and clinical reports.^42^ For permanent and semi-permanent products, lower “retention” estimates may reflect migration from the intended pocket, inflammatory tissue responses, or capsular remodelling that alters contour without necessarily representing true loss of material.^11^, ^12^, ^13^^,^^36^^,^^38^ Importantly, the evidence base for fillers was more heterogeneous in product formulation, measurement approach and follow-up, so interpretation relied predominantly on narrative synthesis rather than balanced head-to-head quantitative comparison across all outcomes.

Aesthetic and patient-reported outcomes generally favoured AFG across reporting studies.^22^, ^25^, ^39^, ^40^ The pooled increase in surgeon-rated “good/excellent” results and higher patient satisfaction scores likely reflect the ability of AFG to create natural contour transitions and texture, with flexibility for iterative refinement during staged sessions. While early physical well-being may favour fillers because AFG involves donor-site liposuction, longer-term satisfaction appeared to favour AFG, consistent with greater durability and fewer clinically significant late adverse events,^11^, ^12^, ^13^^,^^37^ but these findings should be interpreted cautiously because different studies used different surgeon-rating systems, satisfaction scales, and only some used validated instruments such as BREAST-Q. The direction of effect was generally favourable to AFG, but residual measurement heterogeneity limits the strength of quantitative comparison.

Complication profiles differed between interventions, although reporting was not fully standardised. AFG was mainly associated with minor sequelae such as fat necrosis and oil cysts, most of which were managed conservatively, and major surgical intervention was uncommon.^8^^,^^9^ By contrast, permanent and semi-permanent fillers were more often linked to migration, chronic inflammatory reactions, granuloma formation, and higher revision surgery rates that can be more difficult to manage and may require extensive surgical removal.^11^, ^12^, ^13^, ^28^, ^36^ These differences support a more favourable risk–benefit profile for AFG in routine post-reconstructive remodelling, while also highlighting the need for caution when interpreting reoperation data across studies with differing definitions of planned versus unplanned procedures.

Oncological safety remains central in post-oncologic reconstruction. The broader evidence base regarding AFG is reassuring, with pooled hazard ratios approximating unity and no consistent increase in locoregional recurrence reported in large cohorts.^26^, ^29^, ^43^, ^44^, ^45^, ^46^ For fillers, oncologic safety data are comparatively sparse, and additional long-term follow-up studies are required. Radiologic considerations are relevant for both interventions: benign post-AFG changes may prompt further imaging or biopsy, but are generally distinguishable with experienced interpretation, whereas permanent/semi-permanent fillers may introduce persistent imaging artifacts that complicate assessment.^8^^,^^9^^,^^11^, ^12^, ^13^^,^^36^

### Study limitations

The evidence base was limited by heterogeneity in study design, reconstructive context, radiation exposure, staging of procedures, filler formulation, follow-up duration, and outcome definitions. Quantitative synthesis was feasible mainly for AFG volume-retention outcomes; most filler outcomes required narrative synthesis. Several studies were observational and subject to confounding including by radiation status, reconstruction type, recipient-bed quality, timing of remodelling, and staged treatment pathways. Aesthetic and patient-reported outcomes were measured using heterogeneous instruments, and sensitivity analysis restricted to validated tools was not feasible. Reported revision procedures were also inconsistently defined, limiting direct comparison between planned staged AFG sessions and unplanned filler-related reoperations.

Future research should prioritise prospective comparative designs with standardised volumetric measurement protocols, consistent timepoint reporting (including 12 and 24 months), validated patient-reported outcomes, and systematic capture of late adverse events and revision pathways. Comparative studies stratified by filler class and by recipient-bed characteristics (irradiated versus non-irradiated) would be particularly valuable to clarify niche indications and risk–benefit trade-offs.

## Conclusion

Autologous fat grafting is supported by the most consistent evidence for post-reconstructive breast remodelling, particularly with respect to durable volume retention and an acceptable complication profile. Injectable fillers, especially permanent and semi-permanent formulations, were associated with more heterogeneous outcomes and a less favourable late complication profile in the available literature. However, because the evidence base was methodologically heterogeneous and not uniformly comparative across all outcomes, conclusions should remain cautious. Future prospective studies using standardised outcome definitions, validated patient-reported instruments, and clearer reporting of radiation status, reconstructive subtype, and staged procedures are needed.

## Funding

This research received no specific grant from any funding agency in the public, commercial, or not-for-profit sectors.

## Ethics statement

This study is a systematic review and meta-analysis of published literature; therefore, institutional ethics approval and informed consent were not required.

## Declaration of AI and AI-assisted technologies in the writing process

During the preparation of this work, two reviewers screened titles and abstracts, assessed full texts for eligibility and extracted data using a predefined form. All data analysis was conducted by the authors with support from a librarian and statisticians. After the manuscript was drafted, the authors used ChatGPT (OpenAI) only to improve readability and language. No AI tool was used for study screening decisions, data extraction, statistical analysis, or generation of scientific conclusions. The authors reviewed and edited the content as needed and take full responsibility for the content of the publication.

## Declaration of competing interests

The authors declare that they have no known competing financial interests or personal relationships that could have appeared to influence the work reported in this paper.
